# Bone Fragment Granuloma Mimicking a Brain Tumor Following Placement of an Intracranial Pressure Monitoring System

**DOI:** 10.7759/cureus.15394

**Published:** 2021-06-02

**Authors:** Orlando De Jesus, Ricardo J Fernández-de Thomas

**Affiliations:** 1 Neurosurgery, University of Puerto Rico, Medical Sciences Campus, San Juan, PRI

**Keywords:** foreign body, granuloma, icp monitoring, neoplasm, neuroradiology, traumatic brain injury

## Abstract

The placement of intracranial pressure (ICP) monitoring system requires drilling an orifice in the skull. Bone fragments can accidentally be inserted into the brain parenchyma while introducing the ICP monitoring system during the procedure. An intracranial granuloma can be subsequently formed if a non-specific reaction is induced and maintained by the inserted bone fragment in the brain parenchyma. These intracranial granulomas may eventually be confused with brain masses on follow-up imaging studies. We present the case of a 65-year-old male who underwent cranial surgery secondary to a severe traumatic brain injury (TBI). An intracranial bolt was initially placed contralaterally to measure the ICP. Eleven years later, a granuloma from a retained bone fragment secondary to the intracranial bolt placement was suspected. The clinical course, radiological investigations, and differential diagnosis are presented.

## Introduction

The placement of the intracranial pressure (ICP) monitoring system requires perforation of the skull, either with a twist drill craniostomy or a burr hole. During the perforation, bone dust or bone chips may be impacted and retained into the brain at the skull entrance or along the path of the system. These retained bone fragments can produce intracranial granulomas, which may subsequently be confused with brain masses on follow-up imaging studies. We present the case of a 65-year-old man who underwent cranial surgery secondary to a severe traumatic brain injury (TBI) in whom an ICP monitoring system was placed. Several years later, he developed a granuloma from a retained bone fragment that was initially confused as a brain tumor.

## Case presentation

A 65-year-old man was brought to our ED after being found unconscious in the street. His initial neurological examination showed a Glasgow Coma Scale of eight. A head CT scan showed right frontotemporal hemorrhagic contusions with mass effect upon the ipsilateral ventricle and 3 mm right to left midline shift. A left frontal intracranial bolt was placed to measure the ICP. The initial pressure was 26 mmHg, so he immediately underwent surgery to evacuate the hemorrhagic contusions previously depicted in the imaging studies. After a three-week hospital course, he made a slow but uncomplicated recovery and was sent home on antiepileptic medications. Two years later, he returned to the ED after a fall, and a head CT scan with and without contrast was done. Unfortunately, the study was not available; however, the neuroradiologist’s official report indicated a focal round enhancing lesion measuring 7 mm within the left high convexity posterior frontal lobe, which suggested an intra-axial metastatic lesion. However, as the patient did not have a prior history of cancer, and the chest-abdominopelvic CT scan performed was negative, it was believed that it might represent post-surgical changes after the intracranial bolt placement. During the following nine years, he remained stable with no neurological deficits.

**Figure 1:**
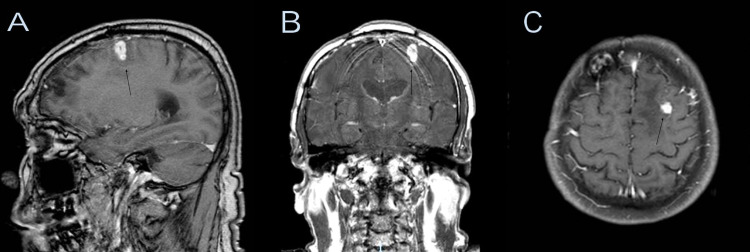
Initial brain MRI with and without contrast showing an intra-axial enhancing mass (black arrow) with associated edema measuring 0.9 x 0.8 x 1.7 cm at the left posterior frontal lobe. Sagittal (A), coronal (B), axial (C).

Eleven years after the trauma, he was brought to our ED due to a seizure and disorientation. A brain MRI study with and without contrast was done, showing an intra-axial enhancing mass with associated edema measuring 9 mm x 8 mm x 17 mm at the left posterior frontal lobe (Figure 1). The lesion was more extensive on the craniocaudal dimension compared to the CT scan performed nine years prior. He had an elevated white blood cell (WBC) count of 11.6 x 109 per liter and a significant urinary tract infection secondary to Escherichia coli. The urinalysis showed many bacteria, positive nitrite, and 20-25 WBC per high power field (HPF). He received intravenous levofloxacin 750 mg daily for five days and was sent home on anticonvulsant medications as the brain lesion was again thought to be reactive.

**Figure 2:**
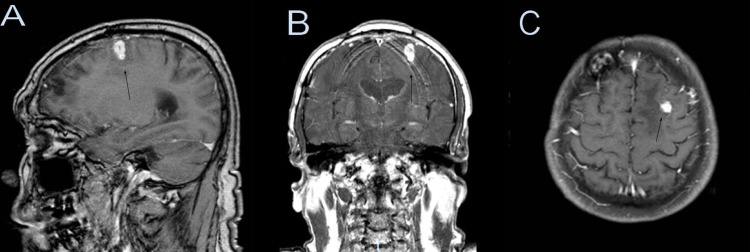
Follow-up brain MRI with and without contrast seven months later, showing that the enhancing mass has reduced (black arrow), measuring 0.7 x 0.7 x 0.7 cm. A central non-enhancing area of 2 mm is noted corresponding to the bone fragment. Sagittal (A), coronal (B), axial (C).

**Figure 3:**
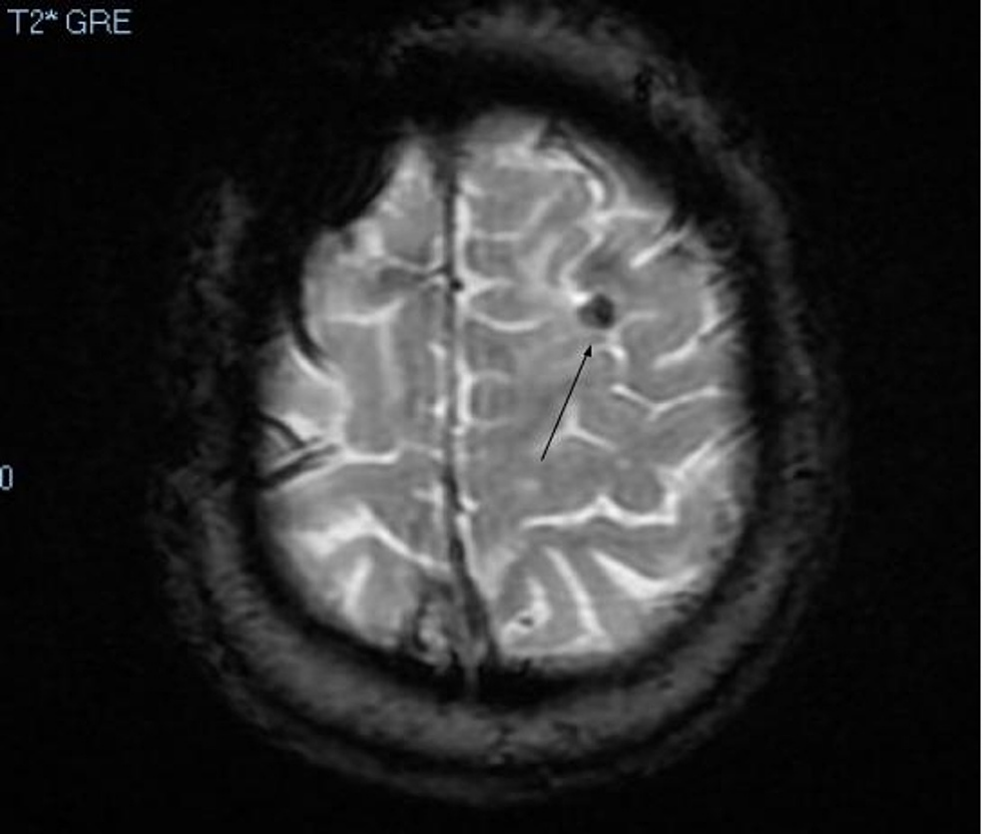
Axial brain MRI gradient echo sequence showing that the lesion (black arrow) appears as a dark lesion, indicating a calcium-containing object.

Seven months later, a repeat brain MRI showed that the enhancing lesion decreased in size and once again measured 7 mm (Figure 2). It was noted that there was a 2 mm central non-enhancing area. The gradient echo (GRE) sequence showed that the lesion appeared as a dark lesion, indicating the possibility of a calcium-containing object as this sequence is highly susceptible to calcium, blood, or iron (Figure 3).

**Figure 4:**
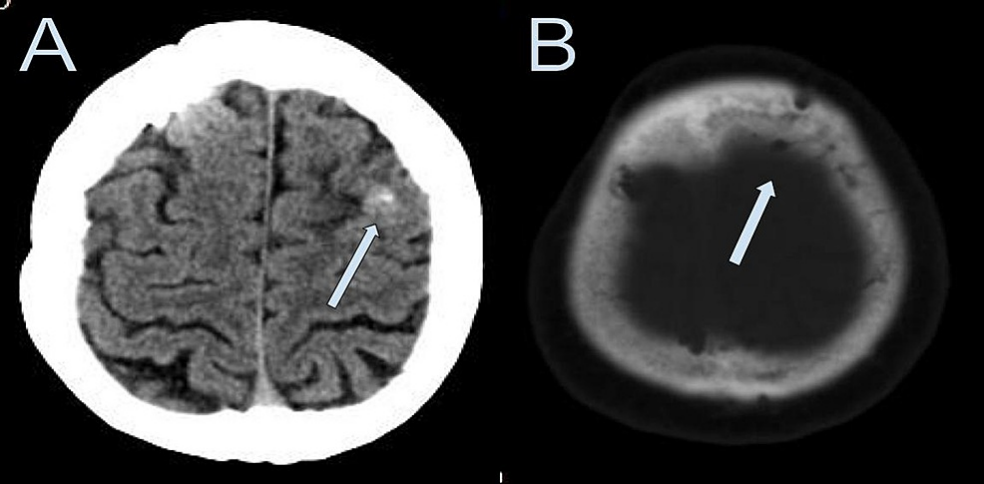
Axial head CT scan performed 15 years after the operation. (A) The small fragment of bone at the granuloma location is shown (white arrow). (B) The bone window image shows the bone defect at the internal and external table of the skull after the twist drill craniostomy (white arrow).

A reactive granuloma secondary to a retained bone fragment was our principal differential diagnosis. We suspect that the lesion enhancement increased secondary to the severe urinary tract infection that the patient developed. We do not suspect that it was an abscess as he was not treated for this and the lesion reduced in size on the follow-up imaging studies performed. We thought that a metastatic lesion is highly improbable as the patient has had the lesion present for more than 13 years. An untreated metastatic lesion having such an indolent course would have been very unlikely. A head CT was repeated four years later, which showed a small fragment of bone at the granuloma location and the bone defect after the twist drill craniostomy (Figure 4). Fifteen years after his trauma, and 13 years after the initial appearance of the left frontal lobe lesion, he remains asymptomatic.

## Discussion

Foreign body granuloma is a chronic, non-specific immune reaction induced and maintained by a foreign material where macrophages are the predominant inflammatory cells [1]. The macrophages may fuse to form multinucleated giant cells involved in the repair processes entailing new vessels and fibrosis formation, which may appear as a mass lesion on contrast-enhanced studies. The cause for such excessive reaction is not clear, but an immunological hypersensitivity of the host against specific antigens is postulated.

Placement of an ICP monitoring system requires drilling an orifice in the skull. Bone fragments can accidentally be inserted into the brain parenchyma while introducing the ICP monitoring system. Bone chips and bone dust have been identified within the path of the ICP monitoring system after its placement and removal [2-5]. Choi et al. found that intracranial bone fragments occur in up to 7% of the cases where a burr hole was used [4]. Using a burr hole instead of a twist drill reduces the chance of introducing the bone fragments into the brain as it allows for direct visualization, irrigation, and cleaning of the area [2].

An intracranial endoscopic procedure is another potential source for inserting bone fragments intracranially. Thomson et al. found that after neuroendoscopy, intracranial calcifications form and gradually enlarge, becoming more clearly defined on each subsequent scan [6]. After their report, the authors stopped using bone dust for packing the skull defect at the end of the procedure. Kafadar et al. also reported a similar complication and recommended not using the bone dust to seal the burr hole [7]. In their case, they performed a lumbar puncture to exclude meningitis, causing the bone dust to be pulled along the endoscope’s track into the ventricular system secondary to the negative pressure gradient created by the lumbar puncture. El Ahmadieh et al. also reported a similar complication after an endoscopic third ventriculostomy (ETV) case with dust migration following a lumbar puncture [8]. They also presented a patient who had migration of the bone dust/fragments covering the burr hole after head trauma. Turhan and Ersahin reported on a patient who had the ETV ostium obstructed by bone dust calcification [9]. Given these reports, it is recommended to place a barrier, such as a pericranium layer or dural substitute in the epidural space between the dura and the bone graft to prevent bone dust from migrating into the endoscopy tract or the ventricles. Intraventricular bone fragments from a prior endoscopy could survive, lead to neovascularization, and grow in the ependyma walls [9]. Bone dust can contain viable osteoblasts capable of dividing and laying down bone when implanted into vascularized tissue. Ji and Ahn found no enlargement of the calcifications between the 12 to 18 months of radiographic follow-up studies [3]. Hence, they postulated that the calcifications enlarged within the first postoperative year, stabilizing after that.

A foreign body inside the brain may form a granuloma that may mimic brain tumors [10]. The foreign body granuloma develops a mass that may enhance after intravenous contrast injection. Usually, these granulomas arise from small pieces of cotton from cottonoids or gauzes after the surgical intervention. Al-Afif et al. described several cases of granulomas with the use of oxidized cellulose polymer [1]. Foreign body granuloma can mimic the recurrence of a brain tumor [11]. Akhaddar et al. were the first to report on a traumatic enhancing granuloma formed a year later after the patient suffered a TBI secondary to a rock impact [12]. However, during surgery, no bone fragment was identified inside the granuloma. Although rare, a foreign body granuloma should be included in the differential diagnosis of intracranial masses or lesions in postoperative patients, especially after a long time interval [1,10,13,14]. The formation of foreign body granuloma associated with absorbable material like oxidized cellulose generally occurred in the first year after the initial operative procedure. In a recent systematic review, the mean time between the primary surgery and the foreign body granuloma diagnosis was 38 months but ranged from 2 weeks to 20 years [13].

On MRI, intracranial foreign body granulomas may appear hypo or isointense in T1-weighted images, hyperintense with a low-intensity ring on T2-weighted images, and brightly enhanced after gadolinium contrast injection [15]. Some authors have suggested obtaining perfusion MRI or spectroscopy to differentiate foreign body granuloma from brain tumors [16]. However, Wang et al. found it unhelpful in their case [17]. In our patient, the brain MRI GRE sequence showed the lesion dark in appearance, indicating the possibility of a calcium-containing object. The granuloma lesion was brightly enhanced after gadolinium contrast injection on the brain MRI but had a central non-enhancing area compatible with the bone fragment. The head CT scan performed 15 years later confirmed the intracranial retained bone fragment.

Cunliffe et al. and Huisman reviewed intracranial lesions mimicking brain tumors; however, none of their cases were caused by granulomas secondary to intracranial bone fragments [18-19]. In a systematic review that included 100 cases of foreign body granuloma after cranial surgery, none were attributed to a bone fragment [13]. Wang et al. were the first to describe a foreign body granuloma secondary to a retained bone fragment from a TBI that occurred 21 years previously, which they initially suspected was a brain tumor [17]. The patient did not receive initial operative treatment, and the wound was only cleaned and sutured. The non-contrast CT scan displayed a high-density lesion with a speckled and bonelike calcification (115 Hounsfield units). Histologic examination of the resected tissue demonstrated that the granuloma was composed of lymphocytes, plasma cells, macrophages, neutrophils, sebaceous glands, and Pacinian corpuscles, with multiple minor abscesses consistent with an immunological reaction.

In our case, the patient required an ICP monitoring device that, during its placement, introduced a bone fragment intracranially, causing a granuloma several years later. We postulate that the enlargement of the enhancing granuloma was secondary to a non-specific inappropriate pro-inflammatory response after the urinary tract infection as its size reduced in the subsequent follow-up imaging study. We believe that in our case, although there is no histological diagnosis, the retained intracranial bone fragment caused the formation of a foreign body granuloma.

## Conclusions

Retained intracranial bone fragments can produce intracranial granulomas. These intracranial granulomas may mimic brain lesions, such as tumors, especially metastatic lesions. Surgery is not indicated unless the granuloma becomes infected, produces a significant mass effect, or causes uncontrolled seizures.
